# Viral interference with SARS-CoV-2 during the COVID-19 pandemic: An individual participant data meta-analysis

**DOI:** 10.1017/S0950268826102088

**Published:** 2026-10-14

**Authors:** Emma S. E. M. Hauser-van Westrhenen, Marieke L. A. De Hoog, Angelique M. A. M. Winkel, Laia Junquera Guinovart, A. H. Ayesha Lavell, Menno D. de Jong, Marlies A. van Houten, Steven F. L. van Lelyveld, Dirk Eggink, Sjoerd Euser, Liesbeth Duijts, Joanne G. Wildenbeest, Rob Schuurman, Janneke H. H. M. van de Wijgert, Margareta Ieven, Katherine Loens, Alike W. van der Velden, Marc J. M. Bonten, Herman Goossens, Patricia C. J. L. Bruijning-Verhagen

**Affiliations:** 1 Department of Epidemiology, Julius Center for Health Sciences and Primary Care, University Medical Centre Utrecht, The Netherlands; 2 Spaarne Gasthuis Academy, Spaarne Gasthuis Hoofddorp, The Netherlands; 3 Department of Medical Microbiology, Amsterdam UMC Locatie AMC, The Netherlands; 4 Department of Internal Medicine, Amsterdam UMC Locatie VUmc, The Netherlands; 5 Amsterdam Institute for Infection and Immunity, The Netherlands; 6 Department of Pediatrics, Spaarne Gasthuis, The Netherlands; 7 Department of Internal Medicine, Spaarne Gasthuis, The Netherlands; 8 National Institute for Public Health and the Environment, The Netherlands; 9 Regional Public Health Laboratory Kennemerland, The Netherlands; 10 Department of Pediatrics and Department of Neonatal and Pediatric Intensive Care, Erasmus University Medical Center Rotterdam, The Netherlands; 11 Department of Pediatric Infectious Diseases and Immunology, Wilhelmina Children’s Hospital, University Medical Centre Utrecht, The Netherlands; 12 Department of Medical Microbiology, University Medical Centre Utrecht, The Netherlands; 13 Laboratory of Medical Microbiology, Vaccine & Infectious Disease Institute, University of Antwerp, Belgium; 14 Department of Primary Care, Julius Center for Health Sciences and Primary Care, University Medical Centre Utrecht, The Netherlands

**Keywords:** SARS-CoV-2, COVID-19, virus infection, viral interference, meta-analysis

## Abstract

Viral interference between SARS-CoV-2 and other respiratory virus infections has been implicated, but symptom-based testing in observational studies may bias estimations. We used individual participant data from ten cohort studies conducted during pandemic years 2020–2024 to study virus interference. Respiratory samples were PCR-tested for viruses including SARS-CoV-2. The primary analysis was based on samples collected from cohort participants regardless of respiratory symptoms. A secondary analysis included only symptom-based testing data. We estimated odds ratios for co-occurrence of SARS-CoV-2 with other respiratory viruses. The primary analysis included data from six cohorts (*n* = 1 023). In total 205 of 2 465 samples were SARS-CoV-2 positive (8.3%). Co-detections with SARS-CoV-2 occurred less frequently than expected under the assumption of zero interference (OR: 0.72; 95% CI: 0.40–1.25) for samples positive for another virus. The secondary analysis (five cohorts, *n* = 1 715) included 2 088 samples from symptomatic subjects, with 506 SARS-CoV-2 infections (24.2%). The OR of SARS-CoV-2 detection was 0.16 (95% CI: 0.10–0.25) for samples positive for another virus. Minimizing selection bias, our results still point towards negative viral interference with SARS-CoV-2, but effect-estimates are weaker and non-significant. Apart from selection bias, the stronger effects found in symptomatic individuals could also indicate that symptomatic infection enhances virus interference.

## Introduction

Following the onset of the COVID-19 pandemic, a sharp decrease in circulation of other respiratory viruses was observed, particularly of respiratory syncytial virus (RSV), influenza virus, human metapneumovirus, and parainfluenza virus. This reduction in detection of respiratory viruses was most likely driven by large-scale non-pharmaceutical interventions such as social distancing and wearing of face masks, which effectively reduced transmission of many viruses in the community [1,2]. Meanwhile, rhinovirus was persistently detected during this period [1,3–5]. The differential effects of the pandemic on circulation of specific respiratory viruses may in part be explained by virus-virus interference. Such interference can be antagonistic or synergistic. Antagonistic interference between heterologous viruses includes those mediated by host innate immune responses. For example, recognition of a virus by pathogen recognition receptors triggers the production of interferons, which subsequently induces and antiviral state in infected cells as well as in neighbouring uninfected cells [6–9]. Conversely, synergistic effects may occur when infection with one virus facilitates subsequent infection with another virus through mechanisms such as enhanced cell fusion or upregulation of cell receptors and adhesion proteins [7]. The nature and magnitude of interference effects likely differ between specific virus pairs. For example, in human airway epithelium cells, concurrent infection of SARS-CoV-2 with RSV reduced replication of RSV, while concurrent infection of SARS-CoV-2 with influenza A did not affect replication of either virus [10].

Now that SARS-CoV-2 has transitioned into an endemic state and circulates along with other respiratory viruses, opportunities for virus-virus interference involving SARS-CoV-2 have increased [11]. Such interference may ultimately impact symptom burden and disease severity. A commonly applied method to study interference between respiratory viruses in epidemiological studies is an observed versus expected approach. This approach compares the number of observed co-detections to the number of expected co-detections under the assumption that virus infections occur independently of each other [12]. To date, studies reported predominantly negative interference of SARS-CoV-2 with other respiratory viruses, including influenza virus, RSV, adenovirus, rhinovirus, parainfluenza virus, metapneumovirus, and seasonal coronaviruses [13–17]. However, these results were largely based on data from healthcare settings, limiting their generalizability to the broader community. Another major limitation is that the collection of respiratory samples in these studies was driven by the presence of symptoms. This approach likely introduces selection bias that results in observed negative interference not necessarily reflecting true antagonism [18,19].

To address these limitations, we analysed data from ten prospective studies during and after the COVID-19 pandemic to evaluate interference. The studies represent various community and healthcare settings, as well as respiratory sampling strategies. Our primary objective was to determine if patterns of viral co-detection indicate existence of viral interference between SARS-CoV-2 and other respiratory viruses, when selection bias arising from sampling criteria is minimized. In addition, to investigate the impact of sampling criteria, we included a secondary analysis based on participants sampled based on the presence of symptoms.

## Methods

### Study design

We conducted an individual participant data meta-analysis on SARS-CoV-2 co-detections with other respiratory viruses, with the primary aim of exploring the impact of co-infection on symptom severity and virus-virus interference. We used individual-level participant data (IPD) from ten COVID-19-related prospective studies spanning a period from March 2020 to May 2024, jointly spanning the entire disease spectrum from asymptomatic community infections to intensive care and fatal cases. These prospective cohorts were included on the basis of IPD access provided through institutional data ownership or existing data-sharing agreements. For a summary of the cohort characteristics, we refer to Supplementary Table S1 and the respective publications [20–27]. Six out of the ten available cohorts applied a respiratory sampling strategy that was not driven by the presence of symptoms. These were household transmission studies as well as individual community cohorts with sampling at fixed timepoints and/or based on virus exposure rather than symptoms. Data from participants were included in the analysis if a (self-collected) respiratory specimen taken regardless of symptoms was available (primary dataset). This included household members of an index case with confirmed SARS-CoV-2 infection and/or acute respiratory symptoms and all participants of the VIOOL cohort. The household members covered ages between zero and 65, whereas the VIOOL participants were all under the age of four. All but two studies collected nose or nose-throat swabs as respiratory specimens. The SARSLIVA household cohorts collected saliva samples.

For our secondary analysis, we used individual-level data from five cohorts comprising participants with symptomatic infection, either from the community or recruited through primary care, where respiratory sampling was performed conditional on presence of symptoms indicating suspected infection. Data were included from symptomatic participants of the CoKids cohort as well as participants enrolled in the community-based cohorts Riding the Waves (RtW) and BCG-Corona who (self-)collected a nose-throat swab upon onset of respiratory symptoms (secondary dataset). Additionally, two primary care cohorts were included (SOS-COVID and MERMAIDS-ARI 1.0) that enrolled and sampled individuals visiting primary care for acute respiratory illness (ARI). All studies were approved by medical ethics committees in the countries where the studies were performed.

All respiratory samples included in the primary and secondary dataset were tested by PCR for a panel of respiratory viruses, including SARS-CoV-2, adenovirus (AdV), bocavirus (HBoV), rhinovirus (HRV), enterovirus (HEV), influenza virus (IFV), respiratory syncytial virus (RSV), human metapneumovirus (HMPV), parainfluenza virus type 1–4 (PIV), and seasonal coronaviruses NL63, HKU1, OC43, and 299E (HCoV) (Supplementary Table S2).

### Variables and definitions

Based on the PCR test results, each sample was categorized as either: (1) virus negative (negative for all viral PCR targets), (2) virus positive for a single virus, and (3) co-detection of two or more viruses. All SARS-CoV-2 positive samples were thus stratified into single or co-detection. For participants who provided multiple samples during follow-up the relevant subset was selected for each analysis to ensure comparability across cohorts. For example, in the VIOOL cohort that employed a weekly sampling schedule, we selected only the first sample collected during a symptomatic episode, while samples taken during asymptomatic periods were all included. Samples were removed from the dataset when the result of SARS-CoV-2 testing and/or respiratory virus testing was unknown. Included samples were further classified according to presence of respiratory symptoms and healthcare setting at the time of sampling into five categories: (1) asymptomatic (no or minimal symptoms), (2) ARI without healthcare attendance, (3) ARI with general practitioner (GP) visit, (4) ARI with hospital admission, and (5) ARI with ICU admission. Comorbidities were categorized as follows: BMI, immunological, cardiological, diabetes, liver, renal, neurological, malignancy, lung, and intestinal. Participants were considered vaccinated against SARS-CoV-2 if at least the primary series was completed, irrespective of time since last vaccination. Influenza vaccination status was not included as this information was not consistently available across all cohorts.

### Statistical analyses

For the primary dataset, characteristics were described for SARS-CoV-2 positive and negative participants. The number of virus detections over time and the most common combinations of respiratory viruses were plotted. Next, we calculated the number of expected co-detections by multiplying the observed number of detections of virus A by the observed number of detections of virus B, divided by the total number of samples, as previously described by Wu et al. [12]. We first calculated this for co-detections of SARS-CoV-2 with any virus and with each virus type separately. This calculation provides the expected number of co-detections under a simple independence model based on marginal frequencies of each virus. The approach does not explicitly account for seasonality of virus circulation and should therefore be interpreted as a descriptive comparison. Next, we used a multivariable logistic regression with detection of SARS-CoV-2 as the outcome and detection of second virus as exposure. The Firth’s bias reduction method was used to account for zero co-detections for some combinations of SARS-CoV-2 with other virus types [28]. We considered the following covariates a priori for inclusion in the model to adjust for the differences between the cohorts and over time: age category (0–5, 6–17, 18–65, and > 65 years), sex, sampling method (saliva or nose(-throat) sample), seasonality, and time period (winter season 2020/2021, 2021/2022, or 2022/2023). We also considered an interaction term for age, but this was not possible due to the low number of SARS-CoV-2 co-detections with some virus types. However, the interaction of age and infection with any virus was not significant. The seasonality term was removed from the final model because of futility. To check the robustness of our results we conducted the following sensitivity analyses: (1) we restricted our analysis to the first sample per participant, omitting multiple events per participant; (2) we repeated the analysis using a mixed-effects logistic regression with cohort as random intercept; and (3) we included seasonality in 2-month intervals.

For the secondary dataset, we applied the same models and covariates with the addition of a parameter for setting (community versus GP). In a sensitivity analysis, we excluded healthcare-based cohorts, as the timing of sample collection in these cohorts was generally later in course of illness compared to participants in community cohorts. This delay may have reduced the likelihood of detecting the virus responsible for the primary onset symptoms.

A Bonferroni correction was applied to account for multiple testing. All analyses were done using R version 4.4.1. and packages logistf 1.26.0 and lme4 1.1–35.4.

## Results

### Primary dataset

The six cohorts covered the period between April 2020 and April 2023. Out of 1023 participants enrolled in this analysis, CoKids (*N* = 358) and SARSLIVA 1.0 (*N* = 201) contributed most participants. The median age of participants included in the analysis was 17 (IQR: 4–41) years, and 46.7% of the participants were female (Table 1). Of the participants, 14.0% had previously received a SARS-CoV-2 vaccination. Most samples were collected during asymptomatic periods (57.5%), followed by ARI without healthcare attendance (39.5%).

**Table 1.** Primary dataset participant characteristics at baseline stratified by SARS-CoV-2 status Table 1. long description.

Characteristic	Overall	SARS-CoV-2 negative	SARS-CoV-2 positive	*P* value
		*n* (%) or median (IQR)		
Participants	*N* = 1 023	*N* = 829	*N* = 194	
Cohort				<0.001
CoronaThuis 1.0	133 (13.0%)	111 (13.4%)	22 (11.3%)	
CoronaThuis 2.0	29 (2.8%)	22 (2.7%)	7 (3.6%)	
SARSLIVA 1.0	201 (19.6%)	109 (13.1%)	92 (47.4%)	
SARSLIVA 2.0	162 (15.8%)	121 (14.6%)	41 (21.1%)	
CoKids	358 (35.0%)	354 (42.7%)	4 (2.1%)	
VIOOL	140 (13.7%)	112 (13.5%)	28 (14.4%)	
Age (median, IQR)	17 (4–41)	17 (4–40)	16 (8–42)	0.31
Age categorical				<0.001
0–5 years	285 (27.9%)	248 (29.9%)	37 (19.1%)	
6–17 years	245 (23.9%)	180 (21.7%)	65 (33.5%)	
18–65 years	493 (48.2%)	401 (48.4%)	92 (47.4%)	
>65 years	0 (0.0%)	0 (0.0%)	0 (0.0%)	
Sex: female	478 (46.7%)	390 (47.0%)	88 (45.4%)	0.67
# comorbidities				0.02
0	704 (69.0%)	577 (69.9%)	127 (65.5%)	
1	287 (28.1%)	222 (26.9%)	65 (33.5%)	
2	27 (2.6%)	26 (3.1%)	1 (0.5%)	
≥3	2 (0.2%)	1 (0.1%)	1 (0.5%)	
Missing	3	3	0	
SARS-CoV–2 vaccination: yes	140 (14.0%)	110 (13.6%)	30 (15.6%)	0.25
Missing	24	22	2	
Samples	*N* = 2 465	*N* = 2 260	*N* = 205	
Cohort				
CoronaThuis 1.0	133 (5.4%)	111 (4.9%)	22 (10.7%)	
CoronaThuis 2.0	29 (1.2%)	22 (1.0%)	7 (3.4%)	
SARSLIVA 1.0	201 (8.2%)	109 (4.8%)	92 (44.9%)	
SARSLIVA 2.0	162 (6.6%)	121 (5.4%)	41 (20.0%)	
CoKids	408 (16.6%)	404 (17.9%)	4 (2.0%)	
VIOOL	1 532 (62.2%)	1 493 (66.1%)	39 (19.0%)	
Disease severity				<0.001
Asymptomatic	1 418 (57.5%)	1 354 (59.9%)	64 (31.4%)	
ARI, no healthcare	974 (39.5%)	843 (37.3%)	131 (64.2%)	
ARI with GP visit	65 (2.6%)	58 (2.6%)	7 (3.4%)	
ARI with hospital admission	7 (0.3%)	5 (0.2%)	2 (1.0%)	
ARI with ICU admission	0 (0.0%)	0 (0.0%)	0 (0.0%)	
Missing	1	0	1	

**Figure 1.**
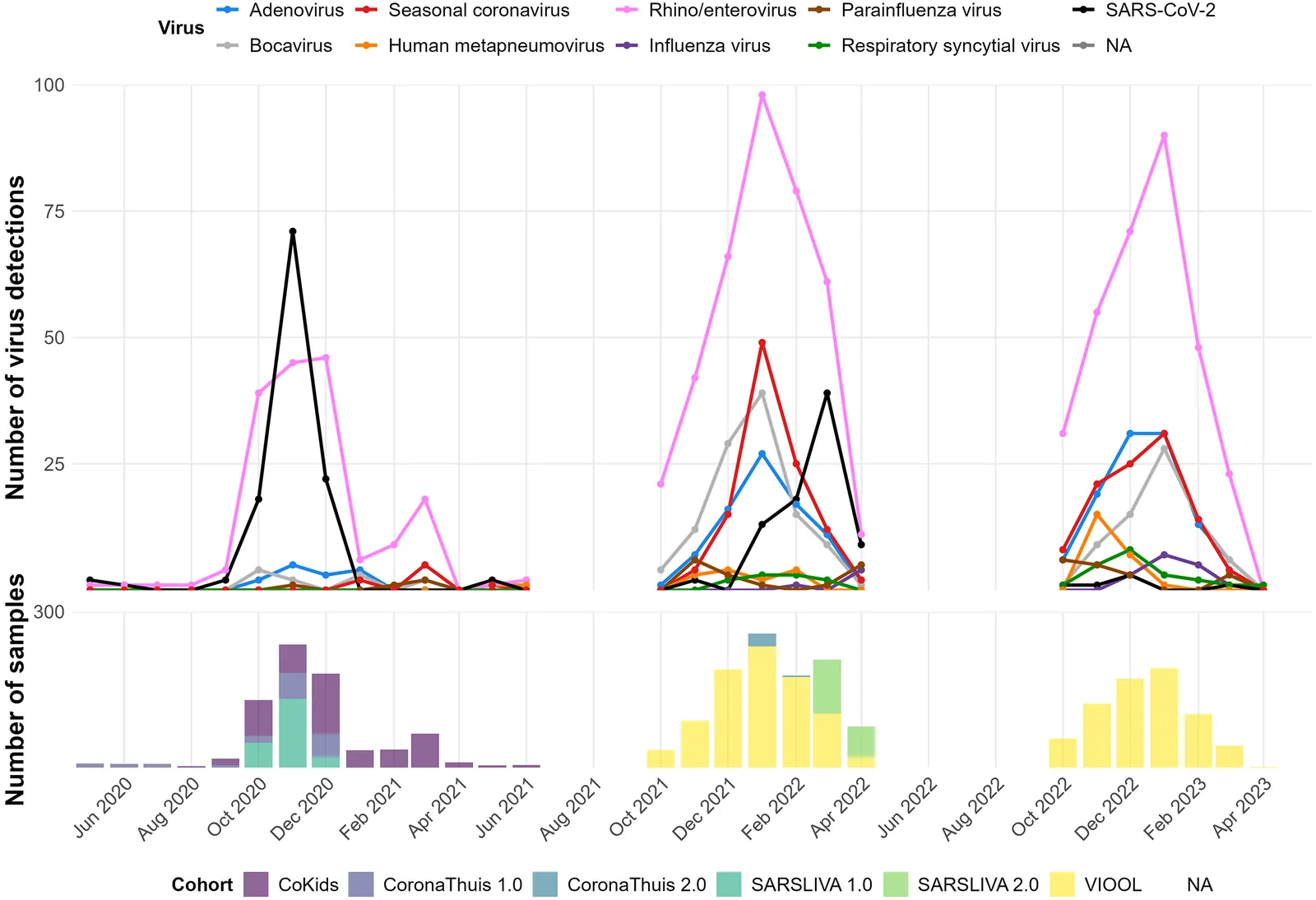
Number of samples collected and respiratory viruses detected over time in the 1023 participants in the primary dataset. Figure 1. long description.

**Figure 2.**
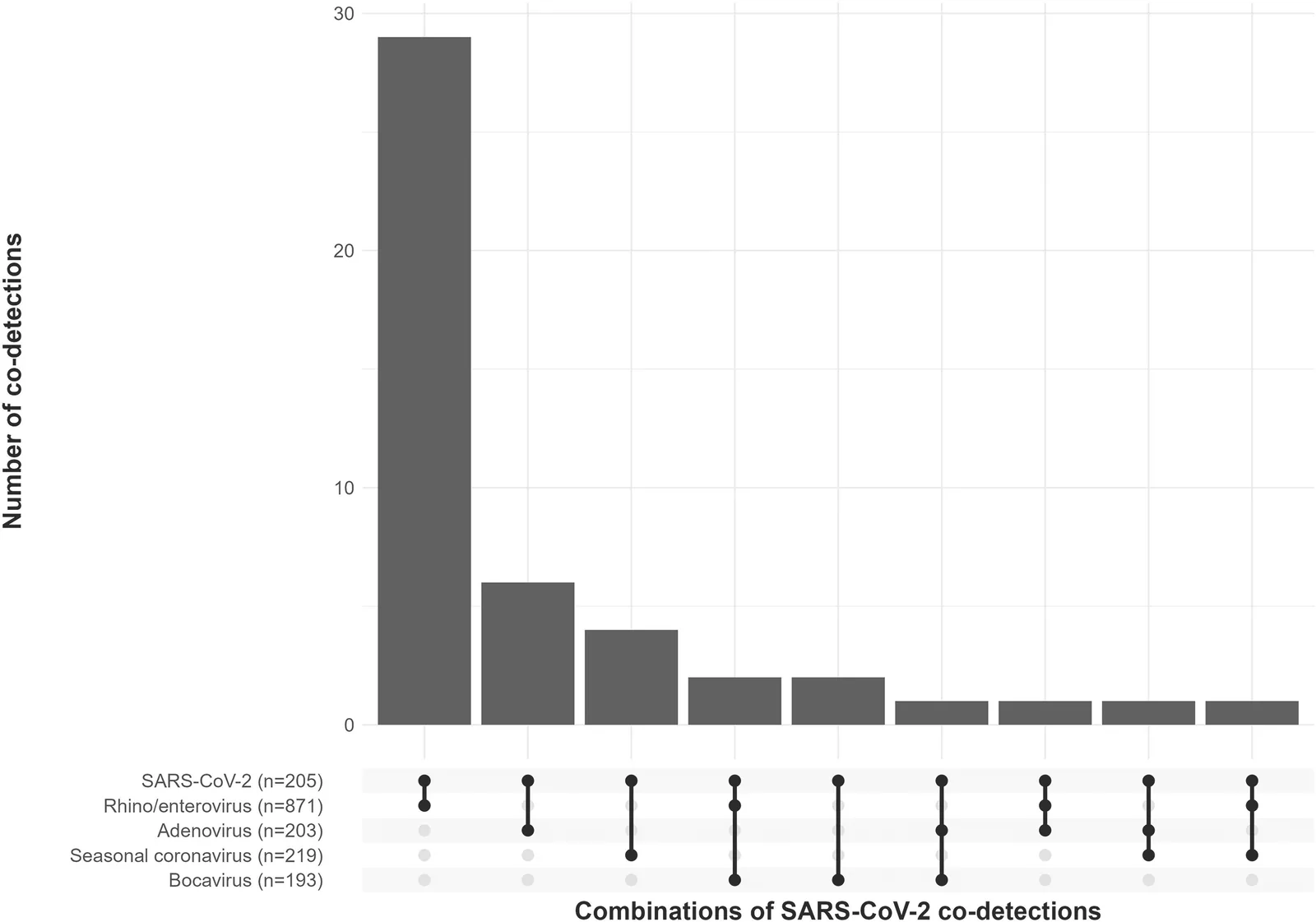
Viral co-detections with SARS-CoV-2 ordered by frequency as detected among 1023 participants in the primary dataset. Figure 2. long description.

In total, 2 465 samples from 1 023 participants were tested for both SARS-CoV-2 and other respiratory viruses. Of these, 205 (8.3%) were positive for SARS-CoV-2, involving 194 participants. Most SARS-CoV-2 infections were detected in the winter of 2020/2021, while detection of other viruses, except rhino/enterovirus, was low during this period compared to subsequent winter seasons (Figure 1). Rhino/enterovirus (*n* = 871, 35.3%) was the most commonly detected non-SARS-CoV-2 virus, followed by seasonal coronaviruses (*n* = 219, 8.9%). Detection of non-SARS-CoV-2 viruses was more common in SARS-CoV-2 negative samples compared to SARS-CoV-2 positive samples. SARS-CoV-2 was most commonly co-detected with rhino/enterovirus (*n* = 33, 16.1% of all SARS-CoV-2 detections) and with adenovirus (*N* = 9, 4.4%; Figure 2).

Co-detections of SARS-CoV-2 with any respiratory virus were observed (*n* = 47) less often than expected under the assumption that the viruses infect independently of each other (*n* = 102). When stratified by virus, all viruses were observed less often in co-detection with SARS-CoV-2 than expected, and no co-detections were found of SARS-CoV-2 with influenza virus, RSV, human metapneumovirus and parainfluenza virus (Table 2). In adjusted logistic regression infection with any virus was associated with 0.72 lower odds of SARS-CoV-2 infection compared to no other virus infection present, but the result was not significant (95% CI: 0.40–1.25). When stratified by virus, most effect estimates pointed towards negative interference, but results were not significant. Sensitivity analysis using a mixed-effects model yielded comparable results, but estimates could not be reliably calculated for virus interference with influenza virus, RSV, human metapneumovirus, and parainfluenza virus because no co-detections were observed (Supplementary Table S3). When restricting the analysis to the first sample per participant results were largely similar, but the low number of co-detections yielded imprecise estimates (Supplementary Table S4). Adjusting for seasonality in 2-month intervals produced broadly consistent estimates, with the exception of influenza and parainfluenza virus, which showed some differences. However, these estimates were based on small numbers and wide, overlapping confidence intervals. The direction of effect remained consistent (Supplementary Table S5).

**Table 2.** Co-detections of SARS-CoV-2 with other viruses in the primary dataset (*N* = 2 465) Table 2. long description.

		Co-detections			
Virus	Detections	Observed	Expected	Unadjusted OR (95% CI^b^)	Adjusted OR^a^ (95% CI^b^)
Any virus	1 225	47	102	0.28 (0.17–0.43)	0.72 (0.40–1.25)
AdV	203	9	17	0.51 (0.17–1.19)	1.84 (0.57–4.84)
HBoV	193	5	16	0.30 (0.07–0.85)	0.91 (0.20–2.83)
HRV/HEV	871	33	72	0.33 (0.19–0.55)	0.66 (0.35–1.20)
IFV	21	0	2	0.25 (<0.001–3.21)	0.46 (<0.001–9.76)
RSV	31	0	3	0.17 (<0.001–2.10)	0.90 (<0.001–12.13)
HMPV	38	0	3	0.14 (<0.001–1.69)	0.61 (<0.001–7.92)
PIV	37	0	3	0.14 (<0.001–1.73)	0.36 (<0.001–5.25)
HCoV	219	6	18	0.31 (0.08–0.82)	0.83 (0.20–2.46)

a Models were adjusted for age, sex, sample type, and time period.

b Bonferroni correction applied: alpha = 0.05/9.

Abbreviations*:* AdV = adenovirus, HBoV = bocavirus, HRV/HEV = rhino/enterovirus, IFV = influenza virus, RSV = respiratory syncytial virus, HMPV = human metapneumovirus, PIV = parainfluenza virus, HCoV = seasonal coronavirus.

### Secondary dataset

In total, 1 715 participants with symptom-based testing were included in the secondary dataset. BCG-Corona, CoKids, MERMAIDS-ARI 1.0 and SOS-COVID were performed between March 2020 and December 2021, while RtW was conducted between October 2022 and May 2024. SOS-COVID was the largest cohort, contributing 855 symptomatic participants. The dataset included slightly more females; the median age was 38 years, and 20.3% had received a SARS-CoV-2 vaccination. Of the 2088 collected samples, most were from participants with an ARI visiting the GP (50.9%) and from participants with an ARI without healthcare attendance (47.4%) (Table 3).

**Table 3.** Cohorts included in the secondary dataset (symptomatic participants) Table 3. long description.

Characteristic	Overall	SARS-CoV-2 negative	SARS-CoV-2 positive	*P* value
		*n* (%) or median (IQR)		
Participants	*N* = 1 715	*N* = 1 218	*N* = 497	
Cohort				<0.001
CoKids	171 (10.0%)	137 (11.2%)	34 (6.8%)	
RtW	359 (20.9%)	224 (18.4%)	135 (27.2%)	
BCG-Corona	219 (12.8%)	135 (11.1%)	84 (16.9%)	
SOS-COVID	855 (49.9%)	660 (54.2%)	195 (39.2%)	
MERMAIDS-ARI 1.0	111 (6.5%)	62 (5.1%)	49 (9.9%)	<0.001
Age continuous	38 (27–52)	37 (25–50)	42 (30–54)	
Age categorical				<0.001
0–5 years	88 (5.1%)	83 (6.8%)	5 (1.0%)	
6–17 years	135 (7.9%)	106 (8.7%)	29 (5.8%)	
18–65 years	1 401 (81.7%)	963 (79.1%)	438 (88.1%)	
>65 years	91 (5.3%)	66 (5.4%)	25 (5.0%)	
Sex: female	1 067 (62.2%)	760 (62.4%)	307 (61.8%)	0.81
# comorbidities				0.53
0	951 (56.3%)	690 (57.1%)	261 (54.4%)	
1	515 (30.5%)	359 (29.7%)	156 (32.5%)	
2	180 (10.7%)	131 (10.8%)	49 (10.2%)	
≥3	42 (2.5%)	28 (2.3%)	14 (2.9%)	
Missing	27	10	17	
SARS-CoV–2 vaccination: yes	314 (20.3%)	182 (16.5%)	132 (29.9%)	<0.001
Missing	169	114	55	
Samples	*N* = 2088	*N* = 1,582	*N* = 506	
Cohort				
CoKids	176 (8.4%)	142 (9.0%)	34 (6.7%)	
RtW	727 (34.8%)	583 (36.9%)	144 (28.5%)	
BCG-Corona	219 (10.5%)	135 (8.5%)	84 (16.6%)	
SOS-COVID	855 (40.9%)	660 (41.7%)	195 (38.5%)	
MERMAIDS-ARI 1.0	111 (5.3%)	62 (3.9%)	49 (9.7%)	
Disease severity				<0.001
Asymptomatic	0 (0.0%)	0 (0.0%)	0 (0.0%)	
ARI, no healthcare	951 (47.4%)	711 (47.0%)	240 (48.5%)	
ARI with GP visit	1 021 (50.9%)	793 (52.4%)	228 (46.1%)	
ARI with hospital admission	31 (1.5%)	7 (0.5%)	24 (4.8%)	
ARI with ICU admission	4 (0.2%)	1 (0.1%)	3 (0.6%)	
Missing	81	70	11	

In total, 2 088 samples of 1 715 participants were tested for both SARS-CoV-2 and other respiratory viruses. SARS-CoV-2 was detected in 506 (24.2%) samples involving 497 participants. The highest number of SARS-CoV-2 infections was detected in winter 2020/2021 (Supplementary Figure S1). Rhino/enterovirus (*n* = 456, 21.8%) was the most commonly detected non-SARS-CoV-2 virus, followed by seasonal coronaviruses (*n* = 127, 6.1%) (Table 4). In 48 of the 506 samples with SARS-CoV-2, a co-detection was observed. SARS-CoV-2 was most commonly co-detected with rhino/enterovirus (*n* = 29, 6.5% of all SARS-CoV-2 detections) and seasonal coronaviruses (*n* = 7, 1.4%) (Table 4 and Supplementary Figure S2).

**Table 4.** Co-detections of SARS-CoV-2 with other respiratory viruses in the secondary dataset (*N* = 2088) Table 4. long description.

		Co-detections			
Virus	Detections	Observed	Expected	Unadjusted OR (95% CI^b^)	Adjusted OR^a^ (95% CI^b^)
Any virus	706	48	171	0.15 (0.09–0.23)	0.16 (0.10–0.25)
AdV	27	4	7	0.59 (0.10–2.11)	0.89 (0.15–3.49)
HBoV	13	4	3	1.48 (0.24–6.80)	2.31 (0.33–13.37)
HRV/HEV	456	29	111	0.17 (0.09–0.28)	0.19 (0.10–0.32)
IFV	48	4	12	0.31 (0.06–1.01)	0.37 (0.07–1.25)
RSV	29	2	7	0.28 (0.02–1.26)	0.37 (0.03–1.72)
HMPV	45	3	11	0.25 (0.03–0.92)	0.38 (0.05–1.42)
PIV	14	2	3	0.62 (0.05–3.26)	1.08 (0.08–7.84)
HCoV	127	7	31	0.18 (0.05–0.46)	0.20 (0.06–0.51)

a Models were adjusted for age, sex, setting, and time period.

b Bonferroni correction applied: alpha = 0.05/9.

Abbreviations*:* AdV = adenovirus, HBoV = bocavirus, HRV/HEV = rhino/enterovirus, IFV = influenza virus, RSV = respiratory syncytial virus, HMPV = human metapneumovirus, PIV = parainfluenza virus, HCoV = seasonal coronavirus.

Similar to the primary dataset results, there were less co-detections of SARS-CoV-2 with any virus than expected. Only bocavirus occurred slightly more often as SARS-CoV-2 co-detection than expected (Table 4). In adjusted analysis, significantly lower odds of SARS-CoV-2 detection were found in samples positive for any virus (OR: 0.16; 95% CI: 0.10–0.25) compared to samples negative for non-SARS-CoV-2 viruses. When stratified by virus type, negative interference effects were significant for rhino/enterovirus (OR: 0.19; 95% CI: 0.10–0.32) and seasonal coronaviruses (OR: 0.20; 95% CI: 0.06–0.51). A sensitivity analysis including only participants from community cohorts showed comparable results with an OR of 0.11 (95% CI: 0.06–0.19) for SARS-CoV-2 infection in the presence of any other respiratory virus (Supplementary Table S6).

## Discussion

In this study we combined data from multiple European cohort studies, jointly covering the entire COVID-19 pandemic period and including both symptomatic and asymptomatic respiratory virus infections to investigate virus-virus interference involving SARS-CoV-2. In our primary analysis of cohorts that applied sampling regardless of the presence of symptoms, all viruses occurred less often as a co-detection with SARS-CoV-2 than expected under a simple independence model. However, viral interference could not be conclusively demonstrated in multivariable analysis despite our large sample size. Our secondary analysis of participants sampled based on symptom presence yielded much stronger effect estimates for negative interference with SARS-CoV-2. When we stratified this analysis by virus type, effect estimates for negative interference were significant for rhino/enterovirus and seasonal coronaviruses. Results were robust in sensitivity analyses when removing repeated events, including a random intercept for cohort or adding a seasonality term to the model.

By utilizing a primary dataset of participants who were sampled regardless of the presence of respiratory symptoms, our study addresses a key limitation of previous studies. Persons with respiratory symptoms may exhibit a different distribution of viral infections than persons without symptoms. Conditioning the analysis on symptom presence increases the likelihood of detecting specific respiratory viruses compared to the general population, and fewer negative samples are found [19]. Moreover, if a symptomatic individual does not test positive for virus X, the likelihood increases that another virus Y is responsible for their symptoms. This mutual exclusivity can result in fewer co-detections than expected under the assumption of independent circulation, erroneously suggesting negative interference [18–29]. Alternatively, if virus X and Y are not individually associated with presence of symptoms, but jointly contribute to symptom development, symptomatic individuals may exhibit a higher rate of X + Y co-detections [18]. Thus, conditioning on symptom status can bias estimates of viral interference in either direction and may yield results that are not generalizable to the wider population.

Our secondary analysis yielded effect estimates of negative viral interference that are in line with those of other cohort studies with symptom-based testing that investigated viral interference using similar statistical approaches. These studies report negative viral interference between SARS-CoV-2 and a variety of other respiratory viruses, including influenza virus, RSV, parainfluenza virus, human metapneumovirus, adenovirus, rhinovirus, and seasonal coronaviruses [13–17]. The stronger negative interference effects observed in ours and other studies based on symptomatic individuals, compared to our primary analysis may reflect the previously described bias. However, biological or behavioural effects may also contribute to the observed differences [18]. Studies in airway epithelium have shown that negative virus-virus interference is likely the result of the expression of interferons and interferon-stimulated genes leading to a temporal anti-viral state [10,30–34]. Possibly, the interferon response is stronger in symptomatic than in asymptomatic infected individuals, thereby increasing the interference effect. A behavioural component could also explain the observed increased negative interference: symptomatic individuals are more likely to self-isolate and stay home, thereby reducing exposure to other circulating viruses. This reduces the likelihood of a second viral infection while the original infection lingers on [35]. Unfortunately, our secondary analysis is not able to distinguish the effect of selection bias from any true difference in interference effect.

The expected co-detection calculation is based on a simple independence model and does not explicitly account for temporal variation in virus circulation. Respiratory viruses have distinct seasonal patterns, and limited temporal overlap between viruses could affect the expected number of co-detections. This effect of distinct seasonality is somewhat limited in our analyses as >85% of samples were collected during the typical respiratory season between October and April. To minimize the possibility of temporal confounding in the association analyses, we a priori considered a seasonality term for the model, but the regression models including seasonality obtained similar results.

A major strength of this study is the joint analysis of individual participant data from multiple cohorts, resulting in a large and comprehensive dataset that spanned the entire COVID-19 pandemic, covered the full spectrum of disease severity and included all age groups. Moreover, all respiratory samples were tested using a broad panel of respiratory viruses, thereby reducing the likelihood of false negative results and enhancing the reliability of co-detection assessments.

Our study has several limitations. First, the use of a single time-point for sample collection does not capture the temporal sequence of co-infecting viruses, nor does it provide information on the duration of the virus being present. Experimental studies in animals and in vitro models suggest the order of virus entry determines both the direction and magnitude of interference between viruses [35]. Furthermore, highly sensitive PCR testing can detect viral RNA or DNA from non-replicating viruses that may persist in human airways for extended periods [36, 37]. In our study, we were not able to distinguish between active infections and prolonged shedding of non-replicating viral genetic material. Second, our mixed-effects models were somewhat oversimplified for estimation purposes and did not fully account for all levels of clustering (household, cohort). Third, although our primary dataset was based on samples collected regardless of symptom presence, the included household members were exposed to an index case with SARS-CoV-2 infection and/or respiratory symptoms. While this likely increased their exposure to the virus that infected the index case when compared to the general population, this exposure is independent of presence or absence of other viral infections in household members and thus our ability to assess interference effects. Most representative for the general population is the VIOOL cohort employing longitudinal weekly sampling, but this cohort only includes young children, limiting generalizability to other age groups. Finally, the circulation of respiratory viruses was profoundly disrupted during the COVID-19 pandemic. Therefore, the interference patterns observed in this study may not fully reflect current dynamics where SARS-CoV-2 and other respiratory viruses co-circulate more consistently.

To conclude, our primary analysis points towards negative viral interference, although non-significant. Stronger and significant effects were found in our secondary analysis of symptomatic individuals. Our study illustrates how negative interference effects become weaker when selection bias from symptom-based testing is minimized. Alternatively, biological effects where symptomatic infection enhances virus interference compared to asymptomatic infection may also contribute to the stronger effects found in our secondary analysis. Jointly, the evidence supports the existence of some negative interference between SARS-CoV-2 and other respiratory viruses but its magnitude and therefore implications for the wider population are difficult to quantify due to methodological challenges. Given the altered circulation patterns of many respiratory viruses during the COVID-19 pandemic, further studies are needed to validate our findings in post-pandemic settings where SARS-CoV-2 and other respiratory viruses co-circulate more consistently.

## Supporting information

10.1017/S0950268826102088.sm001 Hauser-van Westrhenen et al. supplementary material Hauser-van Westrhenen et al. supplementary material

## Data Availability

The individual participant data analysed in this study are not publicly available due to privacy and ethical restrictions. Access to data from the contributing studies may be requested from the corresponding investigators of the original studies, subject to relevant institutional and regulatory approvals.

## Supplementary material

The supplementary material for this article can be found at http://doi.org/10.1017/S0950268826102088.

## Long descriptions

### Table 1. Long description

The table is divided into two main sections: Participants and Samples.

Participants Section (N = 1 023):

- Total participants: 829 negative, 194 positive.

- Cohort distribution: CoKids was the largest cohort for negative participants (42.7 percent), while SARSLIVA 1.0 was the largest for positive participants (47.4 percent). P value less than 0.001.

- Age: Median age was 17 for negative and 16 for positive participants. Age categories showed significant differences (P value less than 0.001), with the 18 to 65 group being the largest in both (approximately 48 percent).

- Sex: 47.0 percent female in the negative group and 45.4 percent in the positive group (P value 0.67).

- Comorbidities: 69.9 percent of negative and 65.5 percent of positive participants had zero comorbidities (P value 0.02).

- SARS-CoV-2 vaccination: 13.6 percent of negative and 15.6 percent of positive participants were vaccinated (P value 0.25).

Samples Section (N = 2 465):

- Total samples: 2 260 negative, 205 positive.

- Cohort distribution: VIOOL provided the most samples overall (62.2 percent).

- Disease severity: For negative samples, 59.9 percent were asymptomatic. For positive samples, 64.2 percent were classified as A R I with no healthcare visit, and 31.4 percent were asymptomatic. P value less than 0.001.

Navigate back to Table 1..

### Figure 1. Long description

The multiplot is divided into two vertical sections sharing a common X axis representing time from June 2020 to April 2023.

Top Panel: A line graph titled Number of virus detections on the Y axis ranging from 0 to 100. It tracks ten categories: Adenovirus, Bocavirus, Seasonal coronavirus, Human metapneumovirus, Rhino or enterovirus, Influenza virus, Parainfluenza virus, Respiratory syncytial virus, S A R S-Co V-2, and N A.

* Period 1 (June 2020 to June 2021): Shows a major peak in S A R S-Co V-2 reaching approximately 70 detections in late 2020, coinciding with a smaller peak in Rhino or enterovirus.

* Period 2 (October 2021 to April 2022): Dominated by a massive peak in Rhino or enterovirus reaching nearly 100 detections in early 2022. Seasonal coronavirus and Bocavirus also show significant peaks around 40 to 50 detections.

* Period 3 (October 2022 to April 2023): Shows a broad peak in Rhino or enterovirus reaching 90 detections, with Adenovirus and Seasonal coronavirus peaking around 30.

Bottom Panel: A stacked bar chart titled Number of samples on the Y axis ranging from 0 to 300. It tracks seven cohorts: CoKids, CoronaThuis 1.0, CoronaThuis 2.0, S A R S L I V A 1.0, S A R S L I V A 2.0, V I O O L, and N A.

* Period 1: Sampling is dominated by S A R S L I V A 1.0 and CoKids, peaking at approximately 250 samples in late 2020.

* Period 2: Sampling is dominated by the V I O O L cohort (yellow), peaking at nearly 300 samples in early 2022.

* Period 3: Sampling is almost exclusively from the V I O O L cohort, peaking at approximately 200 samples in early 2023.

Navigate back to Figure 1..

### Figure 2. Long description

The vertical y-axis is labeled Number of co-detections with increments of 0, 10, 20, and 30. The x-axis is labeled Combinations of S A R S - C o V - 2 co-detections.

Below the bar chart is a matrix with five rows representing viruses and their total sample counts:

1. S A R S dash C o V dash 2 (n=205)

2. Rhino/enterovirus (n=871)

3. Adenovirus (n=203)

4. Seasonal coronavirus (n=219)

5. Bocavirus (n=193)

The data shows a sharp decrease in frequency from left to right:

* The first bar is the highest at 29 detections, showing a co-infection of S A R S - C o V - 2 and Rhino/enterovirus.

* The second bar shows 6 detections for S A R S - C o V - 2 and Adenovirus.

* The third bar shows 4 detections for S A R S - C o V - 2 and Seasonal coronavirus.

* The fourth and fifth bars each show 2 detections. One for a triple infection of S A R S - C o V - 2, Rhino/enterovirus, and Bocavirus; the other for S A R S - C o V - 2 and Bocavirus.

* The final four bars each represent 1 detection for various triple combinations involving S A R S - C o V - 2 with Adenovirus, Seasonal coronavirus, Rhino/enterovirus, and Bocavirus.

Navigate back to Figure 2..

### Table 2. Long description

The table contains six columns: Virus, Detections, Observed Co-detections, Expected Co-detections, Unadjusted O R (95% C I), and Adjusted O R (95% C I).

* Any virus: 1 225 detections; 47 observed; 102 expected; Unadjusted O R 0.28 (0.17–0.43); Adjusted O R 0.72 (0.40–1.25).

* A d V: 203 detections; 9 observed; 17 expected; Unadjusted O R 0.51 (0.17–1.19); Adjusted O R 1.84 (0.57–4.84).

* H B o V: 193 detections; 5 observed; 16 expected; Unadjusted O R 0.30 (0.07–0.85); Adjusted O R 0.91 (0.20–2.83).

* H R V / H E V: 871 detections; 33 observed; 72 expected; Unadjusted O R 0.33 (0.19–0.55); Adjusted O R 0.66 (0.35–1.20).

* I F V: 21 detections; 0 observed; 2 expected; Unadjusted O R 0.25 (<0.001–3.21); Adjusted O R 0.46 (<0.001–9.76).

* R S V: 31 detections; 0 observed; 3 expected; Unadjusted O R 0.17 (<0.001–2.10); Adjusted O R 0.90 (<0.001–12.13).

* H M P V: 38 detections; 0 observed; 3 expected; Unadjusted O R 0.14 (<0.001–1.69); Adjusted O R 0.61 (<0.001–7.92).

* P I V: 37 detections; 0 observed; 3 expected; Unadjusted O R 0.14 (<0.001–1.73); Adjusted O R 0.36 (<0.001–5.25).

* H C o V: 219 detections; 6 observed; 18 expected; Unadjusted O R 0.31 (0.08–0.82); Adjusted O R 0.83 (0.20–2.46).

Models were adjusted for age, sex, sample type, and time period. Bonferroni correction was applied with alpha equal to 0.05 over 9.

Navigate back to Table 2..

### Table 3. Long description

The table is divided into two main sections: Participants (N = 1 715) and Samples (N = 2 088).

Section 1: Participants

- Total: 1 218 negative, 497 positive.

- Cohort Distribution: SOS-COVID is the largest group (49.9 percent overall). P value for cohort distribution is less than 0.001.

- Age: Median age is 38 (IQR 27 to 52). The 18 to 65 years category is the largest (81.7 percent). P value for age categorical is less than 0.001.

- Sex: 62.2 percent are female (P value 0.81).

- Comorbidities: 56.3 percent have 0 comorbidities; 30.5 percent have 1; 10.7 percent have 2; 2.5 percent have 3 or more (P value 0.53).

- SARS-CoV-2 vaccination: 20.3 percent overall are vaccinated (16.5 percent of negatives vs 29.9 percent of positives, P value less than 0.001).

Section 2: Samples

- Total: 1 582 negative, 506 positive.

- Disease Severity: Categorized as ARI (Acute Respiratory Infection). 47.4 percent had no healthcare visit; 50.9 percent had a G P visit; 1.5 percent were hospitalized; 0.2 percent were admitted to the I C U. P value for severity is less than 0.001, with higher hospitalization rates in the SARS-CoV-2 positive group (4.8 percent vs 0.5 percent).

Navigate back to Table 3..

### Table 4. Long description

The table contains six columns: Virus, Detections, Observed Co-detections, Expected Co-detections, Unadjusted O R (95 percent C I), and Adjusted O R (95 percent C I).

* Any virus: 706 detections; 48 observed; 171 expected; Unadjusted O R 0.15 (0.09–0.23); Adjusted O R 0.16 (0.10–0.25).

* A d V: 27 detections; 4 observed; 7 expected; Unadjusted O R 0.59 (0.10–2.11); Adjusted O R 0.89 (0.15–3.49).

* H B o V: 13 detections; 4 observed; 3 expected; Unadjusted O R 1.48 (0.24–6.80); Adjusted O R 2.31 (0.33–13.37).

* H R V / H E V: 456 detections; 29 observed; 111 expected; Unadjusted O R 0.17 (0.09–0.28); Adjusted O R 0.19 (0.10–0.32).

* I F V: 48 detections; 4 observed; 12 expected; Unadjusted O R 0.31 (0.06–1.01); Adjusted O R 0.37 (0.07–1.25).

* R S V: 29 detections; 2 observed; 7 expected; Unadjusted O R 0.28 (0.02–1.26); Adjusted O R 0.37 (0.03–1.72).

* H M P V: 45 detections; 3 observed; 11 expected; Unadjusted O R 0.25 (0.03–0.92); Adjusted O R 0.38 (0.05–1.42).

* P I V: 14 detections; 2 observed; 3 expected; Unadjusted O R 0.62 (0.05–3.26); Adjusted O R 1.08 (0.08–7.84).

* H C o V: 127 detections; 7 observed; 31 expected; Unadjusted O R 0.18 (0.05–0.46); Adjusted O R 0.20 (0.06–0.51).

Adjusted models account for age, sex, setting, and time period. A Bonferroni correction was applied with alpha equal to 0.05 over 9.

Navigate back to Table 4..
