# Supplementary material for: Viral interference with SARS-CoV-2 during the COVID-19 pandemic: An individual participant data meta-analysis
Source: Epidemiol Infect. 2026 Oct 14;154:e128. doi: 10.1017/S0950268826102088 (PMC13749192; doi:10.1017/S0950268826102088)
Supplement: Supplementary file 1 [file S0950268826102088sup001.docx]

**Supplementary Table 1**

*Description of included cohorts*

|  | **Primary analysis** | | | | |  |  | **Secondary analysis** | | | |
| --- | --- | --- | --- | --- | --- | --- | --- | --- | --- | --- | --- |
|  | **CoronaThuis 1.0** | **CoronaThuis 2.0** | **SARSLIVA 1.0** | **SARSLIVA 2.0** | **VIOOL** | **CoK** | **ids^a^** | **RtW** | **BCG-Corona** | **SOS-COVID** | **MERMAIDS-ARI 1.0** |
| **General information** | | | | | | | | | | | |
| **Country** | The Netherlands,  Belgium and  Switzerland | The Netherlands | The Netherlands | The Netherlands | The Netherlands | The Netherlands | | The Netherlands | The Netherlands | Pan-European | Pan-European |
| **Study design** | Household outbreak study | Household outbreak study | Household outbreak study | Household outbreak study | Prospective cohort | Prospective + top-up household outbreak study | | Prospective cohort | Randomized controlled trial | Case cohort study | Case cohort study |
| **Study period** | 4/2020 – 4/2021 | 1/2022 – 4/2022 | 10/2020 – 12/2020 | 3/2022 – 4/2022 | 10/2021 – 5/2022 and  10/2022 – 4/2023 | 8/2020 – 7/2021 | | 10/2022 – 5/2024 | 3/2020 – 4/2021 | 4/2020 – 3/2021 | 3/2020 – 7/2022 |
| **Dominant SARS-CoV-2 variant** | Wild-type, Alpha | Omicron | Wild-type | Omicron | Omicron | Wild-type, Alpha | | Omicron | Wild-type, Alpha | Wild-type, Alpha period | Wild-type, Alpha, Delta, Omicron |
| **Population** | Adults and children | Adults and children | Adults and children | Adults and children | Children <4 years | Adults and children | | Children and adults | Healthcare workers | Children and adults | Adults |
| **Age (median, IQR)** | 33 (13 – 47) | 36 (7 – 39) | 20 (10 – 45) | 21 (12 – 45) | 1 (0 – 2) | 25 (4 – 38) | | 47 (20 – 57) | 41 (30 – 53) | 38 (28 – 50) | 41 (28 – 54) |
| **Sex: female** | 46.6% | 51.7% | 42.3% | 47.5% | 44.3% | 51.5% | | 66.6% | 84.0% | 55.9% | 64.9% |
| **Sample size** | 878 | 315 | 278 | 225 | 143 | 565 | | 359 | 257 | 885 | 945 |
| **Setting** | Community | Community | Community | Community | Community | Community | | Community | Community | GP | GP and hospital |
| **Primary aim** | Study transmission from index case to household members and impact of measures taken in the household | Study transmission from index case to household members and impact of Covid-19 vaccination | Transmission of SARS-CoV-2 within households | Transmission of SARS-CoV-2 within households | Interactions between respiratory viruses | Community incidence and symptom burden of respiratory infections, including SARS-CoV-2 | | Study SARS-CoV-2 immunity and reinfections | Efficacy of BCG vaccine against SARS-CoV-2 | 1) Etiology of RTI’s 2) prediction SARS-CoV-2 3) patient management 4) symptoms associated with SARS-CoV-2 | Identify host and pathogen related determinants of disease severity of ARI of patients presenting in primary of hospital care. |
| **Recruitment** | SARS-CoV-2 screening/testing programs introduced for pandemic control (both in the community and at healthcare facilities) | SARS-CoV-2 screening/testing programs introduced for pandemic control (both in the community and at healthcare facilities) | SARS-CoV-2 screening/testing programs introduced for pandemic control (both in the community and at healthcare facilities) | SARS-CoV-2 screening/testing programs introduced for pandemic control (both in the community and at healthcare facilities) | Random selection children <4 years in the central region of the Netherlands | Households previously participating in 3 Dutch birth cohorts | | Participants from other Covid-19 cohort studies conducted in community, healthcare workers and healthcare setting | Healthcare workers | At primary care upon visit for ARI or suspicion of Covid-19 | At primary care upon visit for ARI |
| **Inclusion criteria** | SARS-CoV-2 positive index case enrolled within 48 hours following PCR test  AND  Household with ≥2 members | SARS-CoV-2 positive index case enrolled within 48 hours following PCR test  AND  Household with ≥2 members of which ≥1 child | SARS-CoV-2 positive index case enrolled within 72 hours following PCR test  AND  Household with ≥3 members | SARS-CoV-2 positive index case enrolled within 72 hours following PCR test  AND  Household with ≥3 members | Daycare attendance and/or an older sibling in the household | Household with ≥2 members of which ≥1 child < age 18 | | Participation in an earlier Covid-19 study | Direct patient contact including Covid-19 patients | Age >1 year with ARI or otherwise suspected of Covid-19 | Patients with ARI presenting to GP or hospital |
| **Follow-up duration** | 21 days or until household outbreak resolved: 21 days after last symptoms onset in a household member | 21 days or until household outbreak resolved: 21 days after last symptoms onset in a household member | 42 days | 42 days | 16 weeks | 23 weeks or less if household completed at most 2 outbreak studies before 23 weeks  For top-up outbreak study: 21 days or until household outbreak resolved: 21 days after last symptoms onset in a household member | | 9 months | 1 year | 28 days | GP: 28 days.  Hospital: until discharge |
| **Sampling methods** | | | | | | | | | | | |
| **Type of biological specimen collected** | Self-collected nose-throat sample | Self-collected nose-throat sample | Self-collected saliva sample | Self-collected saliva sample | Self-collected midturbinate sample | Self-collected nose-throat sample | | Self-collected nose-throat sample | Nasopharyngeal sample | Nasopharyngeal sample | Nasopharyngeal sample |
| **Sampling scheme** | At baseline (irrespective of symptoms) and additionally when a household member developed an ARI ^b^ | At baseline (irrespective of symptoms) and additionally when a household member developed an ARI  additional samples at day 2, day 4, day 7 and day 14 (irrespective of symptoms) | At days 1, 3, 5, 7, 10, 14, 21, 28, 35, 42 | At days 1, 3, 5, 7, 10, 14, 21, 28, 35, 42 | Weekly irrespective of symptoms | Every 4-6 weeks irrespective of symptoms  Top-up outbreak study: at baseline (irrespective of symptoms) and additionally when a household member developed respiratory symptoms ^c^ | | At symptom onset or in case of positive SARS-CoV-2 (self-)test | At symptom onset | At baseline | GP: at baseline and at day 28  Hospital: at baseline, day 28 and at discharge |
| **Samples tested for viral respiratory panel** | Baseline samples | Baseline samples | Baseline samples ^d^ | Baseline samples ^d^ | All samples | Baseline samples | | All samples | Every first positive SARS-CoV-2 sample from a subject along with a matched negative SARS-CoV-2 sample from a subject that remained negative during follow-up | Baseline samples | Baseline samples |
| **Samples selected for analysis** | Baseline samples which were tested for respiratory panel | Baseline samples which were tested for respiratory panel | Baseline samples | Baseline samples | Baseline samples, asymptomatic samples and first sample collected during ARI episode (or <3 days before start ARI) | Outbreak baseline samples from index (SARS-CoV-2 positive and negative) and household members | | All samples | First positive SARS-CoV-2 sample and a negative SARS-CoV-2 sample if the participant didn’t have Covid-19 during follow-up (matched to a positive sample based on intervention, gender and age) | Baseline samples | Baseline samples |
| **Symptom and illness severity measurement** | | | | | | | | | | | |
| **Method** | Daily symptom diary and additional daily severity score during ARI | Daily symptom diary and additional daily severity score during ARI | Symptom diary at saliva collection | Symptom diary at saliva collection | Daily symptom diary and additional daily severity score during ARI | During regular follow up: reporting of new onset respiratory symptoms  During top-up outbreak study: daily symptom diary and additional daily severity score during ARI | | Daily symptom diary from symptom onset or positive SARS-CoV-2 test until day 14 | Daily symptom diary during first 6 months, weekly thereafter | symptom diary at day 0, 7 and 28 | GP reported symptoms at day 0 and 28  Hospital: medical records review |
| **ARI definition** | (1) Onset of fever OR (2) 2 consecutive days with one respiratory and  one systemic symptom or with at least two respiratory symptoms | (1) Onset of fever OR (2) 2 consecutive days with one respiratory and  one systemic symptom or with at least two respiratory symptoms | NA | NA | (1) Onset of fever OR (2) 2 consecutive days with one respiratory and  one systemic symptom or with at least two respiratory symptoms | (1) Onset of fever OR (2) 2 consecutive days with one respiratory and  one systemic symptom or with at least two respiratory symptoms | | NA | NA | NA | NA |

Abbreviations: ARI = acute respiratory illness

^a^ CoKids samples were part of the primary analysis if they were collected irrespective of symptoms, or from the secondary analysis if collected during symptomatic episodes

^b^ No baseline samples from index case at Swiss site

^c^ Outbreak definition: new symptoms or SARS-CoV-2 infection in household

^d^ If baseline sample wasn’t collected or if there was not enough material, then the day 3 sample was tested for respiratory pathogens

**Supplementary Table 2**

*PCR panel and analysed viruses per cohort*

|  | **Primary analysis** | | | | | | **Secondary analysis** | | | | |
| --- | --- | --- | --- | --- | --- | --- | --- | --- | --- | --- | --- |
| **Viruses** | **CoronaThuis 1.0** | **CoronaThuis 2.0** | **SARSLIVA 1.0** | **SARSLIVA 2.0** | **VIOOL** | **CoKids^a^** |  | **RtW** | **BCG-Corona** | **SOS-COVID** | **MERMAIDS-ARI 1.0** |
| SARS-CoV-2 | X | X | X | X | X | X | | X | X | X | X |
| Adenovirus (AdV) | X | X | X | X | X | X | | X | X | X | X |
| Bocavirus (HBoV) | X | X | X | X | X | X | | X | X | X | X |
| Rhino/enterovirus (HRV/HEV) | X | X | X | X | X |  | | X |  | X | X |
| Rhinovirus (HRV) | X | X |  |  | X | X | | X | X | X | X |
| Enterovirus (HEV) | X | X |  |  | X |  | | X |  | X | X |
| Influenza virus (IFV) | X | X | X | X | X | X | | X | X | X | X |
| Respiratory syncytial virus (RSV) | X | X | X | X | X | X | | X | X | X | X |
| Human metapneumovirus (HMPV) | X | X | X | X | X | X | | X | X | X | X |
| Parainfluenza virus type 1-4 (PIV) | X | X | X | X | X | X | | X | X | X | X |
| Seasonal coronaviruses (HCoV) | X | X | X | X | X | X | | X | X | X | X |
| NL63/HKU1 | X | X | X | X | NL63 | X | |  |  | X | X |
| OC43 | X | X | X | X | X | X | |  |  | X | X |
| 229E | X | X | X | X | X | X | |  |  | X | X |

^a^ CoKids samples were part of the primary analysis if they were collected irrespective of symptoms, or from the secondary analysis if collected during symptomatic episodes

**Supplementary Table 3**

*Results sensitivity analysis primary analysis: mixed effects logistic regression (N=2465)*

|  |  | **Co-detections** | |  | |
| --- | --- | --- | --- | --- | --- |
| **Virus** | **Detections** | **Observed** | **Expected** | **Unadjusted OR**  **(95% CI^#^)** | **Adjusted OR^$^**  **(95% CI^#^)** |
| Any virus | 1225 | 47 | 101.88 | 0.65 (0.39 – 1.08) | 0.72 (0.42 – 1.23) |
| AdV | 203 | 9 | 16.88 | 1.46 (0.49 – 3.64) | 1.77 (0.59 – 4.43) |
| HBoV | 193 | 5 | 16.05 | 0.67 (0.15 – 2.03) | 0.73 (0.16 – 2.22) |
| HRV/HEV | 871 | 33 | 72.44 | 0.64 (0.35 – 1.10) | 0.69 (0.38 – 1.22) |
| IFV | 21 | 0 | 1.75 | No co-detections | |
| RSV | 31 | 0 | 2.58 | No co-detections | |
| HMPV | 38 | 0 | 3.16 | No co-detections | |
| PIV | 37 | 0 | 3.08 | No co-detections | |
| HCoV | 219 | 6 | 18.21 | 0.53 (0.13 – 1.56) | 0.60 (0.15 – 1.74) |

^$^ Adjusted for age, gender, sample type and time period, with cohort included as a random intercept

^#^ Bonferroni correction applied: alpha = 0.05/5

Abbreviations: AdV = adenovirus, HBoV = bocavirus, HRV/HEV = rhino/enterovirus, IFV = influenza virus, RSV = respiratory syncytial virus, HMPV = human metapneumovirus, PIV = parainfluenza virus, HCoV = seasonal coronavirus

**Supplementary Table 4**

*Results sensitivity analysis primary analysis: analysis restricted to first sample per participant (N=1023)*

|  |  | **Co-detections** | |  | |
| --- | --- | --- | --- | --- | --- |
| **Virus** | **Detections** | **Observed** | **Expected** | **Unadjusted OR**  **(95% CI^#^)** | **Adjusted OR^$^**  **(95% CI^#^)** |
| Any virus | 331 | 27 | 53.39 | 0.36 (0.19 – 0.65) | 0.78 (0.37 – 1.58) |
| AdV | 33 | 3 | 5.32 | 0.59 (0.08 – 2.25) | 1.94 (0.22 – 9.97) |
| HBoV | 30 | 1 | 4.84 | 0.26 (0.01 – 1.52) | 0.72 (0.02 – 5.90) |
| HRV/HEV | 261 | 20 | 42.10 | 0.36 (0.17 – 0.69) | 0.69 (0.30 – 1.51) |
| IFV | 2 | 0 | 0.32 | 1.04 (<0.001 – 35.33) | 0.74 (<0.001 – 30.13) |
| RSV | 2 | 0 | 0.32 | 1.04 (<0.001 – 35.33) | 8.56 (0.001 – 974.24) |
| HMPV | 8 | 0 | 1.29 | 0.30 (<0.001 – 4.62) | 2.97 (<0.001 – 70.96) |
| PIV | 9 | 0 | 6.61 | 0.27 (<0.001 – 4.00) | 0.74 (<0.001 – 19.35) |
| HCoV | 41 | 4 | 6.61 | 0.61 (0.11 – 2.06) | 1.55 (0.24 – 6.98) |

^$^ Adjusted for age, gender, sample type and time period

^#^ Bonferroni correction applied: alpha = 0.05/9

Abbreviations: AdV = adenovirus, HBoV = bocavirus, HRV/HEV = rhino/enterovirus, IFV = influenza virus, RSV = respiratory syncytial virus, HMPV = human metapneumovirus, PIV = parainfluenza virus, HCoV = seasonal coronavirus

**Supplementary Table 5**

*Results sensitivity analysis primary analysis: seasonality corrected for in 2-month intervals (N=2465)*

|  |  | **Co-detections** | |  | |
| --- | --- | --- | --- | --- | --- |
| **Virus** | **Detections** | **Observed** | **Expected** | **Unadjusted OR**  **(95% CI^#^)** | **Adjusted OR^$^**  **(95% CI^#^)** |
| Any virus | 1225 | 47 | 102 | 0.28 (0.17 – 0.43) | 0.72 (0.40–1.26) |
| AdV | 203 | 9 | 17 | 0.51 (0.17 – 1.19) | 1.78 (0.55–4.73) |
| HBoV | 193 | 5 | 16 | 0.30 (0.07 – 0.85) | 0.97 (0.21–3.06) |
| HRV/HEV | 871 | 33 | 72 | 0.33 (0.19 – 0.55) | 0.63 (0.33–1.16) |
| IFV | 21 | 0 | 2 | 0.25 (<0.001 – 3.21) | 0.94 (<0.001–17.22) |
| RSV | 31 | 0 | 3 | 0.17 (<0.001 – 2.10) | 0.78 (<0.001–10.58) |
| HMPV | 38 | 0 | 3 | 0.14 (<0.001 – 1.69) | 0.58 (<0.001–7.75) |
| PIV | 37 | 0 | 3 | 0.14 (<0.001 – 1.73) | 0.63 (<0.001–8.98) |
| HCoV | 219 | 6 | 18 | 0.31 (0.08 – 0.82) | 0.77 (0.18–2.35) |

^$^ Adjusted for age, gender, sample type and time period in 2-month intervals

^#^ Bonferroni correction applied: alpha = 0.05/9

Abbreviations: AdV = adenovirus, HBoV = bocavirus, HRV/HEV = rhino/enterovirus, IFV = influenza virus, RSV = respiratory syncytial virus, HMPV = human metapneumovirus, PIV = parainfluenza virus, HCoV = seasonal coronavirus

**Supplementary Figure 1**

Number of samples collected and respiratory viruses detected over time in the 1715 participants in the secondary dataset

**Supplementary Figure 2**

*Viral co-detections with SARS-CoV-2 ordered by frequency as detected among 1715 participants in the secondary dataset*

**Supplementary Table 6**

*Results sensitivity analyses secondary analysis: GP cohorts excluded (N=1122)*

|  |  | **Co-detections** | |  | |
| --- | --- | --- | --- | --- | --- |
| **Virus** | **Detections** | **Observed** | **Expected** | **Unadjusted OR**  **(95% CI^#^)** | **Adjusted OR^$^**  **(95% CI^#^)** |
| Any virus | 501 | 26 | 116.99 | 0.09 (0.05 – 0.16) | 0.11 (0.06 – 0.19) |
| AdV | 20 | 2 | 4.67 | 0.44 (0.04 – 2.10) | 0.61 (0.05 – 3.26) |
| HBoV | 5 | 2 | 1.17 | 2.35 (0.15 – 25.92) | 1.95 (0.12 – 25.57) |
| HRV/HEV | 303 | 12 | 70.75 | 0.10 (0.04 – 0.21) | 0.12 (0.05 – 0.26) |
| IFV | 48 | 4 | 11.21 | 0.32 (0.06 – 1.05) | 0.38 (0.07 – 1.27) |
| RSV | 29 | 2 | 6.77 | 0.29 (0.02 – 1.31) | 0.36 (0.03 – 1.68) |
| HMPV | 19 | 0 | 4.44 | 0.08 (<0.001 – 1.05) | 0.08 (<0.001 – 1.09) |
| PIV | 14 | 2 | 3.27 | 0.65 (0.05 – 3.44) | 1.00 (0.07 – 7.01) |
| HCoV | 99 | 4 | 23.12 | 0.14 (0.03 – 0.44) | 0.16 (0.03 – 0.49) |

^$^ Adjusted for age, gender, setting and time period

^#^ Bonferroni correction applied: alpha = 0.05/9

Abbreviations: AdV = adenovirus, HBoV = bocavirus, HRV/HEV = rhino/enterovirus, IFV = influenza virus, RSV = respiratory syncytial virus, HMPV = human metapneumovirus, PIV = parainfluenza virus, HCoV = seasonal coronavirus
